# Hydration Habits and Water Balance in a Working Population in Greece

**DOI:** 10.3390/nu17030371

**Published:** 2025-01-21

**Authors:** Aikaterini-Melpomeni Papadopoulou, Kyriaki Apergi, Margarita-Vasiliki Panagopoulou, Konstantina Argyri, Olga Malisova

**Affiliations:** 1 Department of Food Science and Food Technology, University of Patras, G. Seferi 2, 30100 Agrinio, Greece; aikmelpapado@gmail.com (A.-M.P.); kyapergi@upatras.gr (K.A.); margarita.apanagopoulou@gmail.com (M.-V.P.); 2 Department of Nutritional Science and Dietetics, University of the Peloponnese, Antikalamos, 24100 Kalamata, Greece; kargyri@uop.gr

**Keywords:** water intake, hydration, water balance, cognitive performance, working population

## Abstract

Background/Objectives: Adequate hydration is fundamental for maintaining physical and mental health, yet dehydration remains a common issue, with significant health consequences such as fatigue, impaired cognitive function, and increased risk of chronic diseases. This study aimed to assess the water balance status of healthy employees in Greece and explore the influence of sociodemographic, anthropometric, occupational, and other lifestyle factors. Methods: After completing a validated questionnaire, demographic, dietary, and occupational factors were first analyzed by gender and then examined for their association with water balance, defined as the net difference between water intake and losses, using univariate and multivariable analyses. Results: Daily water consumption had a median value of 3063.77 mL (Q1: 2181.62, Q3: 4070.04), with men consuming significantly more than women. Multivariable analysis identified several factors associated with water balance, including years of education (β: −21.88, SE: 8.55), hours of work per week (β: 30.17, SE: 15.38), use of glasses during the day (Yes vs. No: β: 423.13, SE: 216.41), use of a bottle during the day (Yes vs. No: β: 873.50, SE: 278.82), and consuming water for pleasure (Yes vs. No: β: 478.63, SE: 200.16). Additionally, exposure to weather conditions at work (Yes vs. No) was suggested to have a positive influence on water balance. Conclusions: This study underscores the complex interplay between sociodemographic and occupational factors and hydration practices, providing evidence for targeted hydration strategies, as wellness programs and public health initiatives aimed at improving hydration among working populations in Greece.

## 1. Introduction

Maintaining the body’s water balance is essential for survival and overall well-being, as the body tightly regulates water volume within a narrow range despite daily fluctuations in water intake. Adequate water consumption supports numerous vital functions, including temperature regulation, brain function, kidney health, and metabolic processes [1,2].

Current research highlights the importance of studying hydration as a key factor in achieving optimal health and well-being, shifting the focus beyond athletic performance [3]. Over the past decade, significant advancements in hydration biomarkers have provided valuable insights into their application for health assessments [4,5]. There is robust evidence supporting the benefits of increased water intake for renal health, as well as its connections to broader health outcomes, such as improved skin, gastrointestinal, and cardiometabolic health and the prevention of metabolic dysfunction [6]. Additionally, optimal water intake has been shown to positively influence various aspects of cognitive function, including attention, memory, and decision-making, which are essential for daily performance and well-being [7].

Various factors influence hydration status in workplace settings, with variables such as age, gender, and activity level playing critical roles in determining hydration needs [8,9,10]. In addition, dietary habits—including fluid intake and food choices—can either support or hinder optimal hydration [10,11,12].

In working environments, where individuals often face prolonged periods of physical and mental exertion, the significance of maintaining adequate hydration is often overlooked [7]. The sensation of thirst, a common physiological cue for hydration, is not always a reliable indicator of hydration status. By the time thirst is experienced, dehydration has already occurred, driven by plasma hyperosmolality and hypovolemia—two primary physiological triggers of thirst [1,13].

Employees frequently neglect their hydration needs amidst busy schedules and demanding tasks, leading to dehydration that can negatively affect cognitive function, productivity, and overall well-being. Research shows that even mild dehydration can impair cognitive performance, resulting in difficulties with attention, memory, and decision-making [14]. This decline in brain function can hinder an individual’s ability to perform tasks efficiently, ultimately affecting workplace performance and safety [7].

Furthermore, dietary habits play a critical role in hydration status. Studies have revealed that a large proportion of workers prefer flavored drinks or caffeinated beverages, often containing added sugar or milk, over plain water during work hours. This preference may increase calorie and free sugar intake [15,16], potentially undermining both hydration status and overall workforce health [17].

Understanding the factors that influence hydration in workplace settings is essential for developing effective strategies to promote adequate hydration. Creating an environment that fosters hydration awareness and support can improve employee health, cognitive function, and productivity.

Thus, this study aims to assess hydration status and investigate the various factors influencing it in workplace settings, focusing on demographic, dietary, and occupational determinants in a Greek sample of employees from diverse working environments.

## 2. Materials and Methods

### 2.1. Study Design and Population

In this manuscript, we analyzed data obtained from a cross-sectional survey. Participants had to be healthy adult (18–65 years) employees. The study employed a non-probabilistic convenience sampling method, where participants were selected based on availability and willingness to participate.

The exclusion criteria were: pregnancy, breastfeeding, active urinary tract infections, diagnosed diabetes, kidney diseases, and individuals who have taken medications systematically affecting hydration, or experienced symptoms such as vomiting, diarrhea, or extreme temperature fluctuations in the past 3 days.

Approaching and recruiting potential participants from Greece was conducted through social media outreach and by providing the study link. Each potential participant received a detailed explanation of the study’s objectives and procedures. Informed consent was obtained from all participants before they engaged in the study. The survey, which was conducted in winter for six months, was self-administered via Google Forms. The study received approval from the Bioethics Committee of the University of Patras (number of protocol: 12942, 05-2022).

### 2.2. Questionnaires

To evaluate water balance, we used the Water Balance Questionnaire (WBQ), a validated tool for the Greek population [18]. The WBQ consists of questions of different categories such as (a) demographic socio-economic, (b) work style characteristics—health, (c) physical activity, (d) food and fluid intake, (e) fluid excretion, and (f) trends on fluid and water intake. More specifically, the profile of each employee was outlined through questions on occupation, age, lifestyle, marital status, etc.

Water intake from both solid and liquid foods was recorded using a semi-quantitative drinking frequency questionnaire. To transform the categorical frequency data into a continuous variable, we assigned a mean frequency value to each of the categories based on a reasonable assumption of average consumption within each category. The original beverage consumption variable was measured on a 6-point Likert scale, with categories ranging from “never/rarely” to “>5 times per day”. For recoding, each category was assigned a corresponding mean frequency value: “never/rarely” was assigned 0 times per day, “1–2 times per week” was assigned 0.066 times per day, “3–6 times per week” was assigned 0.214 times per day, “1–2 times per day” was assigned 0.643 times per day, “3–4 times per day” was assigned 1 time per day, and “>5 times per day” was assigned 2.5 times per day. This recoding was implemented using a custom function in R, which mapped each categorical value to its corresponding continuous value. As a result, a new continuous variable was created for each participant, reflecting the mean frequency of beverage consumption, which was then used for further statistical analysis.

The calculation of water from foods included in the questionnaire was based on the data from the USDA National Nutrient Database [19]. Water consumption from water and not from food was calculated separately, using a different variable. Emphasis was given to calculating the intake of fluids such as water, energy drinks, beverages and juices.

For physical activity, the IPAQ questionnaire, short version was used as an evaluating tool [20]. This model includes questions for the last 7 days and focuses on the intensity and duration of each physical activity.

Regarding the loss of fluids, the frequency of urination and defecation were recorded using a 5-point scale. The range of urinary water loss was adjusted based on reports of urinary water loss on a daily basis for healthy individuals [10,21]. A comparable methodology to that utilized for estimating water loss from urination was adopted for the evaluation of water loss from defecation [22]. Sweating was described on a 10-point scale both for resting and exercising time. More specifically, each exercise category (intense, moderate and walking > 10 min) combined with estimated sweating corresponded to a certain amount of fluid loss [10,23,24].

The Likert Scale (1–5) in the questionnaire measures levels of agreement or intensity, with 1 representing the lowest level (e.g., ‘Strongly Disagree’ or ‘Not at all’) and 5 representing the highest level (e.g., ‘Strongly Agree’ or ‘Extremely’). The direction of the scale is clearly defined so that higher values indicate greater strain.

The variable for assessing the variety of beverages consumed by participants was calculated by creating binary variables for each of the five beverage categories included in the questionnaire. A value of 1 was assigned to indicate the consumption of a particular beverage, and a value of 0 was assigned for no consumption. The variable for assessment of the variety of beverages that a participant consumed was calculated by creating new binary variables for each of the five beverage categories included in the questionnaire. To create the categorical variable for beverage variety, a cut-off of 3 was applied based on the median number of beverage types consumed in the study sample. Participants who consumed three or fewer different beverages were categorized as having limited beverage variety (score of 0), while those who consumed more than three beverages were categorized as having broader beverage variety (score of 1).

Water balance was calculated by subtracting the total fluid loss from the total fluid intake in the body. For total fluid intake, we considered: (a) water from foods, (b) water from non-water beverages, and (c) water from drinking water. For total fluid excretion, we included water loss from (a) sweating, (b) urination, and (c) feces. Finally, water lost through respiration (which is incalculable) was equated with metabolic water, as their contributions are nearly equal in quantity but opposite in nature (negative for respiration and positive for metabolic water) [18].

### 2.3. Statistical Analyses

The Shapiro-Wilk test and examination using P-P plots and histograms were used for the assessment of normality. Data are presented as absolute (relative) frequency for categorical variables, mean (standard deviation) or median (Q1–Q3) for continuous variables. The body mass index (BMI) was calculated from the formula: BMI = weight in kg × (Height in meters)^2^. The categorization of BMI, into <18.5 kg/m^2^: Underweight, 18.5–24.9 kg/m^2^: Normal weight, 25–29.9 kg/m^2^: Overweight, ≥30 kg/m^2^: Obesity was based on the criteria of the World Health Organization [25].

For comparisons between genders, the chi-square test was used for categorical variables and *t*-test for independent samples or Wilcoxon rank-sum test for continuous variables.

To investigate the factors associated with fluid balance, univariate and multivariable linear regression analyses were applied. All variables not included in the water balance intake algorithm, but identified as potential confounders or mediators of water balance, based on the current literature and Directed Acyclic Graph (DAG) diagrams, were considered in the analysis.

For multivariable linear regression models, multiple imputation using chained equations (MICE) was employed to address missing data, assuming a Missing at Random (MAR) mechanism. The MAR assumption was assessed using logistic regression models, where a binary indicator for missingness (1 = missing, 0 = observed) was regressed on other observed variables to identify any systematic patterns. Additionally, the plausibility of MAR was evaluated by comparing imputed results with known distributions and logical expectations to ensure consistency.

Variables for the regression models were selected using a combination of theoretical guidance (based on the DAG) and statistical criteria. The full model (Model 1) included all variables identified through the DAG. A backward regression was applied to select variables for the multivariable model using the Akaike Information Criterion (AIC) as the selection criterion (model 2). A reduced model was fitted by including only the variables that were statistically significant at a 5% significance level in model 2 (model 3).

For the multivariable linear regression analyses, we checked for linearity, homoscedasticity, and normality of residuals. Multicollinearity was assessed using the Variance Inflation Factor (VIF).

All statistical analyses were performed using R Core Team (2021). R: A language and environment for statistical computing. R Foundation for Statistical Computing, Vienna, Austria. Available at https://www.R-project.org/, R version 4.1.2 (accessed on 1 November 2021), and RStudio Team (2020). RStudio: Integrated Development for R. RStudio, PBC, Boston, MA. Available at http://www.rstudio.com/ (accessed on 2 November 2024).

## 3. Results

### 3.1. Participant Characteristics 

The study encompassed 208 participants, comprising 98 males and 110 females. In line with standard guidelines for sample size determination in multiple regression, the required sample size for detecting a medium effect size (Cohen’s f^2^ = 0.15) with 80% power and a significance level of 0.05 was calculated to be at least 59 participants for up to 5 predictors. Our final sample size of 208 participants comfortably exceeds this requirement, ensuring robust statistical power for the analyses conducted.

Significant demographic variations emerged, particularly in BMI distribution (*p* < 0.001), where males showed higher proportions in underweight and overweight categories. Residence patterns revealed 60.6% of participants from Athens-Capital, with family status predominantly unmarried (72.6%), displaying notable gender differences (80.4% of males versus 65.85% of females, *p* = 0.060) (Table 1).

### 3.2. Working Environment and Hydration Characteristics

Statistical analysis uncovered significant gender-based differences in labor-intensive work (*p* = 0.019) and weather condition exposure (*p* = 0.012). Participants worked an average of 38.87 h per week, with no significant gender variation. Mental and physical strain levels were relatively consistent across the sample (Table 2).

### 3.3. Water Intake and Discharge

Male and female participants demonstrated distinct water consumption patterns. Males exhibited higher total water consumption (3337 mL/day versus 2912 mL/day, *p* = 0.021) and water extraction (3653 mL/day versus 3015 mL/day, *p* = 0.028). Notably, males consumed more diverse beverages (75.51% versus 60.00%, *p* = 0.013). Both genders had a negative water balance of −327 mL without a statistical difference between them (Table 3).

### 3.4. Factors Affecting Water Balance

The univariate analysis revealed multiple factors influencing water balance with varying statistical significance (Table 4). Several notable predictors emerged across different domains:

Demographic and Personal Characteristics showed minimal direct impact, with gender, age, BMI, and education having limited statistical significance. Notably, specific education variables demonstrated weak correlations with water balance.

Beverage-related factors presented intriguing associations. Coffee/milk/chocolate milk consumption showed a statistically significant positive relationship (b coefficient 371.39, *p* = 0.012), indicating potential metabolic or hydration interactions. Tea consumption (b coefficient 751.62, *p* = 0.004) and alcohol intake (b coefficient 663.83, *p* = 0.016) similarly demonstrated significant correlations with water balance.

Consumption behaviors revealed interesting patterns. Water intake from foods exhibited a strong, statistically significant relationship (b coefficient 1.03, *p* < 0.001), suggesting dietary composition’s crucial role in overall hydration. The consumption of water without thirst and water for pleasure showed moderate positive associations.

### 3.5. Multivariable Analysis

Three progressive regression models systematically explored water balance determinants, maintaining consistent findings with progressively refined predictors (Table 5).

Model characteristics remained stable across iterations, with adjusted R-squared values ranging from 0.09 to 0.10, indicating modest but consistent explanatory power. Root Mean Squared Error (RMSE) remained consistent (1370.04–1377.49).

Consistently significant predictors included years of schooling (negative association, *p* = 0.011–0.017), work hours per week (positive correlation, *p* = 0.051), water bottle usage during the day (strong positive association, *p* = 0.002), and water consumption motivated by pleasure (*p* = 0.018–0.053).

The most robust predictor across models was water bottle usage during the day, with b coefficients around 800–870, suggesting its substantial influence on water balance regulation. All the models explain only a small portion of the variance in water balance, which suggests that other unmeasured factors (e.g., physiological, hormonal, environmental) likely play a significant role. 

## 4. Discussion

Recognizing the global hydration process as integral to health, particularly in professional settings, is vital for fostering an environment that supports both individual well-being and organizational success. Despite its significance, adequate hydration in work environments is often neglected and under-researched. The study provides critical insights into water balance mechanisms, revealing complex interactions between demographic, occupational, and behavioral factors in a sample of working volunteers in Greece. Our findings indicate that the average daily fluid consumption aligned with the recommended hydration guidelines by gender. However, a negative water balance was observed in the overall sample, particularly among women. Notably, the most significant predictor of water balance was the use of water bottles throughout the day, highlighting the importance of convenient access to water for maintaining hydration.

Body water balance depends on the net difference between water gain and water loss. Dehydration occurs when body losses are higher than the intake from liquids and foods, whereas hypohydration refers to the state of water deficit [26]. Less-than-optimal hydration may result in adverse physiological consequences and decreased cognitive performance. A reduction of about only 2% in body mass due to dehydration is consistently associated with greater fatigue feeling along with lower alertness, while severe hypohydration has been found to have detrimental effects on short-term memory and visual perceptual abilities. In contrast, water consumption can improve mood, as well as cognitive performance and visual attention [2]. In our study, the average daily consumption of 3411 mL was consistent with recommended hydration guidelines by gender. However, the negative water balance observed in the overall sample, particularly among women, indicates a potential risk for dehydration, particularly in Greek working populations engaging in physically demanding tasks or high-intensity exercise.

Among the factors that could affect water intake, BMI is considered of major importance, with higher body masses being associated with increased water needs [8,10]. However, in this study involving Greek employers BMI, was not associated with water balance.

The multivariable analysis revealed important predictors of water balance for this working population in Greece. Interestingly, years of education showed a negative association with water intake, meaning that individuals with higher levels of education tended to consume less water. One potential explanation for this finding is that individuals with higher education levels may be more likely to engage in sedentary occupations, where physical activity is limited and opportunities for hydration are fewer throughout the workday. Additionally, higher education levels may be associated with increased workplace stress, which could divert attention away from proper hydration practices. Another factor to consider is the consumption of caffeinated beverages, such as coffee and energy drinks, which are more commonly consumed by individuals with higher education levels. These beverages can have diuretic effects, potentially contributing to lower water balance. While these explanations provide some insight into the observed relationship, further qualitative research is needed to explore these mechanisms in greater depth and confirm the potential factors that mediate this association.

On the other hand, work hours showed a positive correlation with water intake, suggesting that individuals who worked longer hours tended to drink more water. This could reflect increased awareness of hydration needs during extended work periods, or it might be linked to job requirements (e.g., physically demanding jobs or those in environments where regular water intake is encouraged). Additionally, a common trend in workplaces is having something to drink on the desk while working, which encourages regular hydration, especially in jobs with sedentary tasks or long durations where workers are more likely to sip water frequently throughout the day.

In this study, the consumption of specific beverages had distinct effects on water balance. Increased consumption of coffee/milk, tea, and alcohol (during the day) appeared to be positively associated with water balance, meaning that higher intake of these beverages correlated with better hydration status. This association was statistically significant, suggesting that these drinks may contribute to overall fluid intake, potentially influencing hydration levels. Even though coffee and tea contain caffeine, which has mild diuretic effects, studies suggest that their hydrating properties outweigh the diuretic effect when consumed in moderate amounts [8]. Milk, too, is rich in water and can contribute significantly to hydration. While alcohol is typically associated with dehydration, moderate alcohol consumption (particularly in the context of social or structured environments) may not necessarily lead to dehydration if accompanied by water or other non-alcoholic beverages [8]. Finally, in some settings, such as offices or social environments, coffee and tea are commonly consumed alongside water. This could indicate that people who regularly consume these drinks also have access to and drink more water as part of their daily routine, influencing their overall hydration.

On the other hand, having a greater variety of beverages (more than three different types) did not show a significant association with water balance. The analysis found no meaningful relationship between the diversity of beverages consumed and hydration status (b coefficient = 126.76, *p*-value = 0.571), suggesting that simply drinking a wider range of beverages does not necessarily lead to improved water balance. This implies that the type and quantity of beverages consumed may have a greater impact on hydration than the number of different drinks included in a person’s diet.

The study reveals that except for plain water, beverages—including coffee, milk, tea, and alcohol—significantly influence hydration dynamics, supporting optimal physiological functioning in the working population in Greece. Employers have a critical role in promoting workplace hydration through comprehensive strategies. Providing clean drinking water, encouraging water consumption over sugary or caffeinated alternatives, and supporting dietary choices rich in water-content foods can create meaningful interventions. This observation aligns with the existing literature suggesting that male employees often have lifestyle habits that contribute to higher body weight [27], potentially influencing their hydration needs. These approaches extend beyond simple hydration, potentially influencing cognitive function, workplace performance, and overall employee well-being.

The use of water bottles during the day, and not only during working hours, and water consumption for pleasure emerged as robust predictors, underscoring the importance of personal hydration strategies beyond traditional workplace interventions. Model 3 (Table 5), while not statistically significant overall, demonstrated a notable influence on the prediction of water balance. This suggests that while the model may not significantly improve the overall prediction accuracy, it contributes to the overall understanding of the factors influencing water balance. Specifically, the consistent significance of water bottle usage during the day highlights its substantial impact on water intake, emphasizing the importance of hydration strategies in daily life.

The use of water bottles both in working environments and outside the workplace, positively correlated with improved hydration, highlighting the importance of personal hydration strategies. This underscores the importance of accessible hydration tools, like the use of bottles, in promoting adequate fluid intake, particularly in workplaces where physical activity is prevalent. The findings emphasize that addressing hydration must consider conditions both in the working environment and beyond the workplace to encompass lifestyle choices and individual behaviors.

In this study, several factors influencing hydration practices were assessed, including occupational specifics and environmental contexts. For example, labor intensity and exposure to weather conditions were measured using a Likert scale (1–5), allowing us to capture the potential influence of these factors on hydration behavior. Occupational factors, such as labor intensity (e.g., manual labor vs. desk jobs), are crucial, as they can significantly impact fluid requirements due to physical exertion and increased fluid loss through sweat. Similarly, environmental factors, such as exposure to weather conditions (e.g., working outdoors versus in air-conditioned settings), can influence hydration needs due to variations in temperature and heat exposure. Although these factors were considered in our analysis for this working population in Greece, a deeper exploration of their role in hydration practices is warranted and future research should investigate how occupational and environmental contexts, as reflected in these scales, contribute to water balance and hydration behaviors.

The research underscores the importance of viewing hydration as a holistic health strategy. By creating environments that prioritize fluid intake and nutritional awareness, organizations can develop more sophisticated approaches to employee wellness. Water consumption is not merely about meeting basic physiological needs, but about creating comprehensive strategies that enhance mental clarity, reduce fatigue, and support long-term health outcomes. Implementing targeted hydration interventions—such as strategically placed water stations, scheduled hydration breaks, and educational programs—can transform workplace culture. These approaches recognize that employee health is a complex interplay of nutritional choices, workplace environment, and individual behaviors.

The potential benefits extend beyond immediate performance improvements, potentially contributing to reduced healthcare costs, increased productivity, and enhanced workforce engagement. The synergy between balanced nutrition and adequate hydration represents a critical, yet often overlooked, component of workplace wellness strategies. By adopting a comprehensive approach that considers individual variations in hydration needs, organizations can create more supportive, health-conscious work environments that benefit both employees and overall organizational performance.

Despite the rigorous control of the current study’s protocol, several limitations must be acknowledged. Firstly, due to the cross-sectional nature of the study, no causal relationships can be formulated. While the study provides important correlations between factors and water balance, causality cannot be established and future longitudinal or experimental research is needed to establish causal links. Another potential limitation of this study is the possibility of misreporting the dietary intake that may arise from the use of the FFQ. Furthermore, self-reported data, particularly regarding physical activity and dietary habits, may be influenced by memory bias or social desirability bias. Future studies should incorporate objective measures of hydration status alongside self-reports. Also, data collection occurred during winter, which may not fully capture hydration needs during warmer months. Future research could examine seasonal variations and their effects on hydration. While multiple imputation was used to address missing values under the Missing at Random (MAR) assumption, it is important to consider that any deviation from this assumption could affect the robustness of the results. Future studies should aim to collect complete data whenever feasible to minimize this limitation and ensure that findings are not biased by the imputation process. We acknowledge the potential limitations of using social media for participant recruitment, as it can result in a convenience sample that may not perfectly represent the broader Greek workforce. Social media recruitment may introduce biases related to health awareness, education, and urban/rural residence. Despite these potential biases, social media provides a cost-effective and efficient means of reaching a diverse and broad population, which is particularly important given the study’s focus on workplace hydration. To mitigate this risk, we made efforts to ensure that our sample represented various professional sectors and geographic areas within Greece. Specifically, participants were recruited from diverse professional backgrounds, including public and private employees, self-employed individuals, and those working in both labor-intensive and desk-based jobs. Additionally, the sample included participants from both urban and rural regions of Greece, reflecting varying environmental conditions and cultural practices that could influence hydration behaviors.

Finally, while this study provides valuable insights into hydration practices and water balance in the workplace, the non-random sampling method limits the generalizability of our findings. As a result, the sample may not fully represent the broader working population in Greece. This limitation must be considered when interpreting the findings. Future studies should aim to use probabilistic or quota-based sampling methods to improve the generalizability of the results.

## 5. Conclusions

In conclusion, our findings highlight the multifactorial determinants of water balance in the Greek working population, emphasizing the importance of both workplace and individual factors in hydration strategies. Notably, water bottle usage emerged as a robust predictor of hydration, underscoring the role of personal habits in maintaining adequate water intake. Employers can foster hydration by providing accessible resources, promoting water-rich diets, and encouraging balanced hydration practices. Future research should focus on longitudinal designs to explore causal relationships and develop tailored interventions for diverse populations.

## Figures and Tables

**Table 1:** Demographic and body mass characteristics of a sample of volunteers.

	Total (n = 208)	Males (n = 98)	Females (n = 110)	*p*-Value
Gender	208	98 (46.6%)	110 (53.4%)	
Residence				
Athens-Capital	126 (60.6%)	63 (64.9%)	63 (58.8%)	0.208
Other city	68 (32.7%)	29 (29.9%)	39 (35.1%)	
Small Town	10 (4.8%)	5 (5.2%)	5 (4.5%)	
Village	4 (1.9%)	0 (0%)	4 (3.6%)	
Family status				
Unmarried	151 (72.6%)	78 (80.4%)	73 (65.85)	0.060
Married	47 (22.6%)	16 (16.5%)	31 (27.9%)	
Divorced	10 (4.8%)	3 (3.1%)	7 (6.3%)	
Profession				
Self-employed	29 (13.9%)	10 (10.3%)	19 (17.1%)	0.085
Private Sector Employee	76 (36.5%)	31(32.0%)	45 (40.5%)	
Public Sector Employee	27 (6.8%)	10 (10.3%)	17 (15.3%)	
Other (contractor/intern etc)	76 (36.5%)	47 (47.5%)	29 (27.1%)	
ΒΜΙ (kg/m^2^)	24.0 (17.27)	25.89 (25.06)	22.37 (3.39)	0.182
BMI				
<18.5 kg/m^2^	26 (6.5%)	19 (19.6%)	7 (6.3%)	**<0.001**
18.9–24.9 kg/m^2^	114 (28.6%)	34 (35.1%)	80 (72.1%)	
25.0–29.9 kg/m^2^	44 (11.0%)	28 (28.9%)	12 (14.4%)	
>30.0 kg/m^2^	17 (4.3%)	12 (12.4%)	5(4.5%)	

BMI: Body Mass Index; Values are presented as absolute frequency (percentage) for categorical variables or as mean (standard deviation) for continuous variables. *p*-values for comparisons between men and women were calculated using the chi-square test for categorical variables and the independent samples *t*-test for continuous variables. Statistical significant values are highlighted in bold.

**Table 2:** Descriptive data on the working environment and hydration-related benefits.

Working Hours per Week	Total (n = 208)	Males (n = 98)	Females (n = 110)	*p*-Value
	38.87 (19.7)	38.88 (29.84)	40.47 (6.61)	0.368
Labor-intensive				
1	86 (41.30%)	42 (43.3%)	44 (39.6%)	**0.019**
2	31 (14.9%)	10 (10.3%)	21 (18.9%)	
3	41 (19.7%)	14 (14.4%)	27 (24.3%)	
4	31 (14.9%)	17 (17.5%)	14 (12.6%)	
5	19 (9.1%)	14 (14.4%)	5 (4.5%)	
Physical strain				
1	56 (26.9%)	29 (29.9%)	27(24.3%)	0.265
2	50 (24.0%)	21 (21.6%)	29 (26.1%)	
3	59 (28.4%)	23 (23.7%)	36 (32.4%)	
4	30 (14.4%)	15 (15.5%)	15 (13.5%)	
5	13 (6.3%)	9 (9.3%)	4 (3.6%)	
Mental strain				
1	15 (7.2%)	7 (7.2%)	8 (7.2%)	0.973
2	18 (8.7%)	9 (9.3%)	9 (8.1%)	
3	52 (25.1%)	26 (26.8%)	26 (23.4%)	
4	71 (34.1%)	32 (33.0%)	39 (35.1%)	
5	52 (25.0%)	23 (23.7%)	29 (26.1%)	
Access to restrooms				
1	14 (6.7%)	8 (8.2%)	6 (5.4%)	0.280
2	11 (5.3%)	5 (5.2%)	6 (5.4%)	
3	20 (9.6%)	13 (13.4%)	7 (6.3%)	
4	35 (16.8%)	18 (18.6%)	17 (15.3%)	
5	128 (61.5%)	53 (54.6%)	75 (67.6%)	
Exposure to weather conditions				
1	129 (62.0%)	57 (58.8%)	72 (64.9%)	**0.012**
2	35 (16.8%)	17 (17.5%)	18 (16.2%)	
3	25 (12.0%)	12 (12.4%)	13 (11.7%)	
4	8 (3.8%)	1 (1.0%)	7 (6.3%)	
5	11 (5.3%)	10 (10.3%)	1 (0.9%)	
Access to water/beverages at work				
1	10 (4.8%)	7 (7.2%)	3 (2.7%)	0.297
2	5 (2.4%)	3 (3.1%)	2 (1.8%)	
3	28 (3.5%)	16 (16.5%)	12 (10.8%)	
4	39 (18.8%)	15 (15.5%)	24 (21.6%)	
5	126 (60.6%)	56 (57.7%)	70 (63.1)	
Use of water bottle at work				
Yes	115 (55.3%)	51 (52.6%)	64 (57.7%)	0.462
Use of water bottle during the day				
Yes	179 (84.8%)	87 (88.8%)	91 (82.7%)	0.298
Use of glass during the day				
Yes	144 (69.3%)	66 (31.7%)	75 (36.1%)	0.998

The Likert Scale ranges from 1 (lowest) to 5 (highest) indicating varying levels of agreement or intensity; Values are presented as absolute frequency (percentage) for categorical variables. *p*-values for comparisons between men and women were calculated using the chi-square test for categorical variables. Statistical significant values are highlighted in bold.

**Table 3:** Data on water intake and discharge.

Variable	Total	Females	Males	*p*-Value
n	208	110 (52.89%)	98 (47.11%)	
Water From Foods (mL/day)	462 [291, 688]	415 [280, 672]	505 [306, 733]	0.112
Water From Beverages (mL/day)	174 [91, 287]	183 [112, 289]	160.09 [77, 272]	0.216
Juices/Soft Drinks/Soda (times/day)	0.07 [0.07, 0.21]	0.07 [0.02, 0.21]	0.07 [0.07, 0.21]	0.912
Coffee/Milk/Chocolate Milk (times/day)	0.21 [0.07, 0.64]	0.64 [0.07, 0.64]	0.21 [0.07, 0.64]	0.267
Milkshake/Granita (times/day)	0.00 [0.00, 0.00]	0.00 [0.00, 0.00]	0.00 [0.00, 0.00]	NA
Tea (times/day)	0.07 [0.00, 0.21]	0.07 [0.00, 0.21]	0.07 [0.00, 0.21]	0.805
Alcohol (times/day)	0.07 [0.00, 0.21]	0.07 [0.07, 0.21]	0.07 [0.00, 0.21]	0.133
Beverages Variety (>3 Different Types)	140 (66.35%)	66 (60.00%)	74 (75.51%)	**0.013**
Water From Water (mL/day)	2240 [1495, 3185]	2240 [1470, 2698]	2415 [1703, 3650]	0.057
Water Total Consumption (mL/day)	3064 [2182, 4070]	2912 [2170, 3758]	3337 [2392, 4396]	**0.021**
Water Extraction Total (mL/day)	3278 [2625, 4227]	3015 [2536, 4035]	3653 [2657, 4454]	**0.028**
Water Balance (mL/day)	−328 [−1122, 917]	−367 [−1047, 645]	−283 [−1211, 1198]	0.533

Values are presented as absolute frequency (percentage) for categorical variables and median values [Q1. Q3] for continous variables; *p*-values for comparisons between men and women were calculated using the chi-square test for categorical variables and Wilcoxon rank-sum test for continuous variables. Statistical significant values are highlighted in bold.

**Table 4:** Results of univariate regression analysis on factors that affect water balance.

Variable	b Coefficient	Standard Error	*p*-Value	Adjusted R-Squared
Gender (Males)	126.76	223.43	0.571	0.002
Age (years)	196.22	213.54	0.359	0.005
BMI (kg/m^2^)	−0.50	8.67	0.954	0.000
Education (school years)	−19.16	25.82	0.459	0.003
Profession Self-employed	62.29	450.6	0.734	0.011
Private Sector Employee	−242.74	397.99		
Public Sector Employee	−377.84	464.57		
Hours of work per week	17.78	16.69	0.289	0.007
Labor-intensive (Likert scale 1–5)	5.95	78.66	0.940	0.000
Physical strain (Likert scale 1–5)	22.95	89.86	0.799	0.000
Mental strain (Likert scale 1–5)	−79.58	93.69	0.397	0.004
Access to WC at work (Yes)	−68.08	88.01	0.440	0.003
Exposure to weather conditions at work (Yes)	124.16	93.97	0.188	0.009
Access to water at work (Yes)	−8.22	102.21	0.936	0.000
Use of water bottle at work (Yes)	90.39	214.08	0.673	0.001
Beverages types				
Juices/soft drinks/soda (times/day)	−67.50	384.33	0.861	0.000
Coffee/milk/chocolate milk (times/day)	371.39	146.88	**0.012**	0.033
Milkshake/Granita (times/day)	−154.66	1970.14	0.938	0.000
Tea (times/day)	751.62	256.03	**0.004**	0.044
Alcohol (times/day)	663.83	272.67	**0.016**	0.031
Beverages variety (>3 different types)	126.76	223.43	0.571	0.002
Use of glasses during the day (Yes)	171.09	225.93	0.450	0.003
Use of bottle during the day (Yes)	802.49	293.51	**0.007**	0.038
Consumption of water without feeling thirsty (Yes)	356.46	235.12	0.131	0.012
Consumption of water for pleasure (Yes)	454.65	214.50	**0.035**	0.023

The Likert Scale ranges from 1 (lowest) to 5 (highest) representing the degree of agreement or intensity for each statement, with 1 indicating “Strongly Disagree” or “Very Low” and 5 indicating “Strongly Agree” or “Very High”; For gender, “Females” is the reference category; for Profession, the reference category is “Other” (e.g., contractor/intern, etc.); for Beverages variety, the reference category is “<3 different types”; for Yes/No variables, the reference category is “No”; The *p*-value for the b coefficient(s) from the univariate linear regression analysis derived using the *t*-test for significance. Statistical significant values are highlighted in bold.

**Table 5:** Results of multivariable regression analysis about factors that affect water balance.

	Variable	b Coefficient	Standard Error	*p*-Value	Adjusted R Squared
model1	(Intercept)	−2763.38	1333.34	0.040	0.06
	Beverages variety (>3 different types)	165.95	218.21	0.448	**RMSE**
	Gender (Males)	153.30	258.58	0.554	1343.13
	Age (years)	7.16	9.33	0.444	
	BMI (kg/m^2^)	−34.93	29.49	0.238	
	Education (school years)	**−19.66**	**9.10**	**0.032**	
	Profession Self-employed	7.98	446.72	0.986	
	Private Sector Employee	−378.45	401.68	0.347	
	Public Sector Employee	−582.54	453.93	0.201	
	Hours of work per week	**36.82**	**17.54**	**0.037**	
	Labor-intensive (Likert scale 1–5)	−23.06	115.37	0.842	
	Physical strain (Likert scale 1–5)	−32.53	129.00	0.801	
	Mental strain (Likert scale 1–5)	−22.76	92.99	0.807	
	Access to WC at work (Yes)	−82.08	102.39	0.424	
	Exposure to weather conditions at work (Yes)	168.05	106.05	0.115	
	Access to water at work (Yes)	127.49	120.84	0.293	
	Use of water bottle at work (Yes)	117.08	208.24	0.575	
	Use of glasses during the day (Yes)	400.12	227.77	0.081	
	Use of bottle during the day (Yes)	**791.47**	**296.15**	**0.008**	
	Consumption of water without feeling thirsty (Yes)	−31.21	258.28	0.904	
	Consumption of water for pleasure (Yes)	**458.55**	**235.57**	**0.053**	
model2	(Intercept)	−3237.44	807.10	0.000	0.10
	Education (school years)	**−20.66**	**8.57**	**0.017**	**RMSE**
	Hours of work per week	**30.10**	**15.33**	**0.051**	1370.04
	Exposure to weather conditions at work (Yes)	127.38	85.41	0.137	
	Use of glasses during the day (Yes)	**432.78**	**215.86**	**0.046**	
	Use of bottle during the day (Yes)	**855.25**	**278.26**	**0.002**	
	Consumption of water for pleasure (Yes)	**454.88**	**200.20**	**0.024**	
model3	(Intercept)	−3052.46	799.89	0.000	0.09
	Education (school years)	**−21.88**	**8.55**	**0.011**	**RMSE**
	Hours of work per week	**30.17**	**15.38**	**0.051**	1377.49
	Use of glasses during the day (Yes)	**423.13**	**216.41**	**0.052**	
	Use of bottle during the day (Yes)	**873.50**	**278.82**	**0.002**	
	Consumption of water for pleasure (Yes)	**478.63**	**200.16**	**0.018**	

RMSE: Root Mean Squared Error; The Likert Scale ranges from 1 (lowest) to 5 (highest) representing the degree of agreement or intensity for each statement, with 1 indicating “Strongly Disagree” or “Very Low” and 5 indicating “Strongly Agree” or “Very High”; For gender, “Females” is the reference category; for Profession, the reference category is “Other” (e.g., contractor/intern, etc.); for Beverages variety, the reference category is “<3 different types”; for Yes/No variables, the reference category is “No”; Model 1 includes all variables identified through the DAG; Model 2 was derived using backward stepwise regression with the Akaike Information Criterion (AIC); Model 3 includes only variables statistically significant at the 5% level from Model 2; The *p*-value for the b coefficient(s) from the multivariable linear regression analysis derived using the *t*-test for significance. Statistical significant values are highlighted in bold.

## Data Availability

The data presented in this study are available on request from the corresponding author. The data are not publicly available due to the confidential nature of some information.
